# Transdiagnostic investigation of schizophrenia and autism spectrum: Heart rate variability changes during rest, relaxation, and cognitive tasks

**DOI:** 10.1192/j.eurpsy.2025.615

**Published:** 2025-08-26

**Authors:** F. Kinga, Á. Vass, Z. Pálffy, G. Csukly

**Affiliations:** 1 Department of Psychiatry and Psychotherapy, Semmelweis University; 2 Department of Cognitive Science, Budapest University of Technology and Economics, Budapest, Hungary

## Abstract

**Introduction:**

Autism spectrum disorder (ASD) and schizophrenia (SCH) are distinct diagnoses. However, they share common features such as high heritability, neurodevelopmental origins, and difficulties in social communication. Despite this, the etiology and precise pathophysiology of both conditions remain unclear, and no biomarker can definitively distinguish between them. In addition to higher-level cognitive and social-communication skills, autonomic regulation capacity is crucial for emotional regulation in social situations, which has been shown to differ in ASD and SCH according to the literature.

**Objectives:**

Our study, part of a larger research project, aimed to explore autonomic regulation in ASD and SCH by investigating heart rate (HR) and heart rate variability (HRV) as key markers of autonomic nervous system functioning. We measured these parameters during rest, relaxation (body scan), and cognitive tasks to assess changes in autonomic regulation capacity across different conditions.

**Methods:**

Participants underwent an electrocardiogram (ECG) recording during a longer EEG experiment. We analyzed heart rate and HRV data from 114 participants (*N*_ASD_= 38, *N*_SCH_= 37, and *N*_NTP_= 39), with a particular focus on the RMSSD parameter as a key marker of parasympathetic regulation. We hypothesized that HRV would be lower in ASD and SCH groups compared to neurotypical controls (NTP), with the differences between groups diminishing during tasks. The experimental setup avoided additional stressors outside of the social context of the study.

**Results:**

As hypothesized, we found significant differences in RMSSD between groups during the initial resting state (eyes open *F*(2,111)=6.314, *p*=0.003, *ηp2*=0.102, eyes closed (EC): *F*(2,98)=6.800, *p*=0.002, *ηp2*=0.122). Although HRV was nominally lower in the ASD group (EC: *M*_ASD_= 26.10), only the SCH group (EC: *M*_SCH_= 19.77) showed a significant difference from the NTP group (EC: *M*_NTP_= 32.78) based on post hoc comparisons. Contrary to expectations, HRV did not significantly change in the SCH and NTP groups during tasks. However, in the ASD group, HRV increased after body scan relaxation and, notably, during cognitive tasks (group main effect in repeated measures ANOVA: *F*(2,105)=6.068, *p*=0.003). This suggests that the structured nature of the task may have a calming effect, observable in autonomic regulation.

**Conclusions:**

Our findings indicate distinct autonomic regulation patterns in ASD and SCH, with structured situations potentially having a calming effect, particularly in individuals with ASD. In the next phase of our research, we will systematically examine the relationship between electrophysiological parameters and key symptoms such as attachment insecurity, mentalization deficits, disorganization, and the severity of clinical symptoms, all of which have significant implications for clinical conditions and everyday functioning.

**Disclosure of Interest:**

None Declared

